# To tape or not to tape: annular ligament (pulley) injuries in rock climbers—a systematic review

**DOI:** 10.1186/s13102-022-00539-6

**Published:** 2022-08-01

**Authors:** Robin Larsson, Lena Nordeman, Christina Blomdahl

**Affiliations:** 1 Din Hälsocentral Kilafors, Primary Health Care, Region Gävleborg, Kilafors, Sweden; 2 Unit of Physiotherapy, Department of Health and Rehabilitation, Institute of Neuroscience and Physiology, The Sahlgrenska Academy, University of Gothenburg, Gothenburg, Sweden; 3 Research, Education, Development and Innovation, Primary Health Care, Region Västra Götaland, Borås, Sweden

**Keywords:** Pulley injuries, Ligament injuries, Finger injuries, Rock climbing, Taping, Rehabilitation, Conservative treatment, Sports medicine

## Abstract

**Background:**

Popularity of rock climbing is steadily increasing. With its inclusion in the Olympic Games this will likely continue. Injuries from rock climbing are also increasing. The most common injury is to the flexor pulley system, consisting of the finger flexors and five annular ligaments (pulleys). Treatment of this injury includes taping of affected fingers, but evaluation of this treatment was previously lacking. The aim of this review was therefore to assess whether taping is associated with better outcomes than non-taping. A secondary aim was to present treatment recommendations or areas for future research.

**Methods:**

Systematic searches of PubMed, Scopus, SPORTDiscus, Cochrane Library, PEDro and CINAHL. Free text searches of Google Scholar. Citation searching. No restrictions to language, date of publication or study design. Included studies were assessed using Cochrane scale for clinical relevance, by two independent authors. Results were presented in narrative synthesis. Certainty of evidence (GRADE) was assessed by three authors. Review was done according to PICO-protocol and reported according to PRISMA-guidelines.

**Results:**

After removing duplicates, 595 records were identified. Eight studies and one case report (in nine articles, one poster) were included, consisting of 206 rock climbers, four non-climbers, 23 pairs of cadaver hands. Clinical relevance ranged from 0 to 5 (median 2). Evidence of low to moderate certainty suggests that taping might reduce bowstringing of the finger flexor tendons by 15–22%. Evidence regarding pain, time for return to sports, shearing forces against pulleys, pulley ruptures and maximum voluntary contraction (MVC) were all regarded as “very low”, “very low to low” or “low”, and were not considered reliable. Evidence of moderate certainty suggests that taping has no effect on MVC or muscle activation in uninjured rock climbers. No adverse effects of taping were reported.

**Conclusion:**

Low to moderate evidence suggests that taping might reduce bowstringing of the finger flexor tendons. Moderate evidence suggests that taping has no effect on MVC or muscle activation in uninjured climbers. For other outcomes more studies evaluating the effects of taping are needed.

*Trial registration*: PROSPERO CRD42021241271, date of registration: 18-04-2021.

## Background

Rock climbing as sport and recreation has seen a steady increase in popularity during the last years. At the same time, injuries from rock climbing and training for rock climbing have increased as well [1]. This trend is also likely to continue, with rock climbing now being included in the Olympic Games for the first time in Tokyo 2020/2021 [2]. This means that an understanding of the biomechanics of climbing and climbing related injuries are becoming increasingly more important for physiotherapists, sports physicians, hand surgeons, occupational therapist and other healthcare professionals that assess and treat these injuries.

Rock climbing differ from most other sports in that a majority of all injuries affect the fingers and hands, accounting for a total of 42–65% of all climbing related injuries [3, 4]. Of these, injuries to the flexor pulley system are the most common, and account for 15–20% of total injuries [4]. The flexor pulley system consists of the tendons of the two finger flexors, flexor digitorum superficialis (FDS), that inserts on the base of the middle phalanges 2–4, and flexor digitorum profundus (FDP), that inserts on the base of the distal phalanges 2–4, as well as their tendon sheaths. It also consists of a string of ligaments that holds these tendons in place against the phalanges: five annular ligaments/pulleys (A1-5, listed from proximal to distal) and three cruciate ligaments/pulleys (C1-3, listed from proximal to distal). These ligaments prevent bowstringing of the flexor tendons, i.e. distancing of the tendons from the phalanges during flexion of the finger. Of these, the A2- and the A4-pulleys are the strongest and therefore, in this aspect, the most important ones [5]. They are also the two most commonly injured in rock climbing [4].

The aetiology of the almost unique injuries to the flexor pulley system in rock climbing are due to the anatomy of the hand and fingers (described above), its biomechanical properties and the forces that rock climbers habitually put on these structures. In rock climbing, the A2-pulley is regularly exposed to forces of up to 380 N [6], but can reach forces of 450 N if suddenly shock loaded, as for example during a foot slip [7]. As a comparison, 10 Newton is roughly the equivalent of one kilogram (kg), meaning that rock climbers habitually load a single annular ligament of a single finger with loads of around 40 kg.

Grip position also plays a major role. In rock climbing, one of the following is commonly used: open hand, half crimp or full crimp. During open hand the metacarpophalangeal joints (MCP) and the proximal interphalangeal joints (PIP) are fully extended and only the distal interphalangeal joints (DIP) are flexed. This gives biomechanical conditions for very low loads on the pulleys [8]. During half crimp the MCP-joints are slightly flexed, the PIP-joints in 90° flexion and the DIP-joints straight or slightly hyper-extended, this greatly increases the load on the pulleys, especially A2/A4. During full crimp the MCP-joints are in 60° flexion, the PIP-joints in 90° flexion and the DIP-joints maximally hyper-extended [9], this sets the condition for maximum loads on the A2/A4-pulleys [8], with forces on the A2 of up to 36 times that of open hand [10].

Injuries to the annular ligaments, i.e. pulley-injuries, can be both acute, e.g. during a single high intensity overload, or as a result of continued persistent overuse [11]. To classify the severity of pulley-injuries Schöffl et al. [11, 12] have proposed a four-score grading system that is presented below (Table 1), and that will be used in the following to differentiate between the different types of pulley injuries.

**Table 1:** Pulley-injury score, closed injuries

Grade	Injury	Treatment
1	Pulley strain	Conservative (tape)
2	Complete rupture of A4 or partial rupture of A2 or A3	Conservative (tape)
3	Complete rupture of A2 or A3	Conservative (thermoplastic ring + tape)
4	Multiple ruptures (as A2/A3, A2/A3/A4) or single rupture (as A2 or A3) combined with lumbricalis muscle or collateral ligament trauma	Surgical reconstruction

Modified from Schöffl et al. [11] and used with permission

Schöffl et al. [11, 12] have also proposed treatment strategies for these injuries, where grade 1–3 is treated conservatively and grade 4 with surgical reconstruction. The conservative treatment strategy consists of immobilisation for up to 2 weeks (grade 2–3) followed by functional training and use of a thermoplastic ring (grade 3) or tape (grade 1–2). The use of thermoplastic rings for grade 3 injuries have then been further supported by later research [13, 14], but falls without the scope of this review. Time to return to sport has been estimated to be six to eight weeks (grade 1–2) and three months (grade 3), with continued use of protective taping for three months (grade 1–2) or six months (grade 3).

Supportive taping of the fingers have thereafter been used frequently by rock climbers, but few attempts have been made to evaluate this approach [15]. Results have also been contradictory, and only one systematic review, in Slovenian, has been published to date [16], including a total of four studies [6, 9, 15, 17].

The aim and purpose of this systematic review was therefore to identify and analyse the available research on finger taping for rock climbers, as part of conservative treatment of pulley injuries (grade 1–3), to assess whether taping is associated with better outcomes than non-taping. A secondary aim was to use these data to present treatment recommendations for conservative treatment of pulley injuries in rock climbers. Or, if sufficient data is lacking, suggest future research necessary for such recommendations.

## Methods

### Protocol and registration

A protocol for this systematic review was registered in PROSPERO (PROSPERO 18-04-2021: CRD42021241271). The review was conducted according to the PICO-process [18, 19] and reported according to the Preferred Reporting Items for Systematic Reviews and Meta-Analysis statement (PRISMA) [20].

### Eligibility criteria and PICO

We used the PICO-framework to form eligibility criteria. PICO = Population, Intervention, Comparisons, Outcomes [18, 19].

*Population*: rock climbers with annular ligament (pulley) injuries and/or risk of these injuries, i.e. men and women of all ages who injured or not injured themselves during rock climbing and/or training for rock climbing. Due to rock climbing being a relatively new sport with little published material, non-climbing subjects (including cadavers) with annular ligament (pulley) injuries independent of aetiology, as well subjects with risk of these injuries, were also eligible for inclusion.

*Intervention*: any method of supportive taping of the annular ligaments (pulleys) of the finger.

Comparison: no supportive taping of the annular ligaments of the finger. Presence of a control group was not a necessary condition for inclusion.

*Outcomes*: pain; function (functional rating scales); time to return to sport (RTS); bowstringing, i.e. distance of the finger flexor tendon from the phalange (mm); maximum voluntary contraction (MVC) of the finger flexors (FDS/FDP); force (N) against the annular ligaments (pulleys); maximum force at annular ligament (pulley) rupture; and/or any indirect measure of these outcomes.

*Exclusion criteria*: primary treatment consisting of thermoplastic cast/ring, splints or orthopaedic fixation devices; surgical reconstruction; invasive therapy (e.g. corticosteroid injections); review article or other non-original research.

No restriction was otherwise set to study design, sample size or methodology.

### Literature search

We developed a search strategy together with input from librarians at the Biomedical Library Gothenburg University, Sweden. A search strategy was developed for PubMed, then subsequently adapted to the other databases. The search strategy combined search terms with medical subject headings and comprised combinations, synonyms and variants of “pulley”, “annular ligament”, “finger tendon”, “climbing”, “athletic injury” and/or “tape” (“Appendix 1”). Searches were conducted in the databases PubMed, Scopus, SPORTDiscus, Cochrane Library, PEDro and CINAHL, in March 2022. We also searched Google Scholar for grey literature, in October 2021, using free text variants of above search terms. We also contemplated using Google's standard search engine, but decided against it, since our target was unpublished study results and original data, not secondary sources. Finally we searched ClinicalTrials.gov for ongoing studies, and performed backward and forward citation searches of included studies, and identified previous reviews, for additional relevant records. We did not apply any restrictions to language or date of publication.

### Study selection and data extraction

Identified records were imported into Rayyan (a web and mobile app for systematic reviews) [21] for screening, after removing duplicates using EndNote [22]. Any additional identified duplicates were removed manually. All three authors (RL, CB and LN) screened identified titles and abstracts, and when necessary full text articles, for inclusion independently (according to eligible criteria). Records were marked as either “included”, “excluded” or “maybe” independently. The authors were not blinded to trial identifiers such as authors’ and journals’ names. Disagreements and records marked as “maybe”, as well as all retrieved full texts, were discussed among all three authors until consensus was reached. To assess agreement among reviewers, percentage agreement and Cohen’s kappa was calculated.

Two authors (RL and CB or LN) performed data extraction independently. Extracted data included study design, participant demographics (including age and sex), intervention components for experimental and control group, outcome measures and outcome data.

### Clinical relevance and certainty of evidence assessment

Two authors (RL and LN) assessed included studies independently for relevance using the Cochrane scale for clinical relevance. [23]. A mean difference of < 10% was considered a small effect size; 10–20% medium; and > 20% big [24]. Studies on cadavers automatically lost one point due to not being directly comparable to living individuals seen in practice. Any disagreements were resolved by discussion among all three authors until consensus was reached. To assess agreement among reviewers, percentage agreement and Cohen’s kappa was calculated. We also contemplated assessing the studies using the PEDro scale for quality [25], but found it unmerited, due to the large heterogeneity in study design, including both case reports and cohort studies as well as clinical trials and randomized controlled trials. The clinical relevance score was then used, together with other criteria, in the overall assessment of the certainty of the evidence (GRADE).

We assessed the certainty of evidence using the Grading of Recommendations Assessment, Development and Evaluation (GRADE) approach, using the following criteria: risk of bias, inconsistency, indirectness, imprecision, and reporting bias [26]. Results from controlled trials were initial assigned a certainty level of four (high), results from observational studies a certainty level of two (low) and results from case reports a certainty level of one (very low). Certainty of evidence was then rated down half a point, one point or two points if we detected issues with risk of bias, inconsistency, indirectness, or imprecision. Publication bias was not assessed due to the small number of studies, and their heterogeneous results, but was not considered likely. We saw no reason to rate up the level of evidence neither due to large effect size, dose–response effect nor effects of residual confounding factors.

### Data synthesis and analysis

Characteristics of included studies were synthesised in relevant tables and charts, when deemed appropriate, and analysed in a narrative synthesis. Due to the large heterogeneity between included studies, in regards to population, intervention and outcome measures, a meta-analysis of the data was not possible. When possible, missing p-values were calculated from available means, standard deviations (SD) and sample sizes. When numerical values were missing altogether, these were estimated visually from available figures/diagrams. When necessary, corresponding authors were contacted directly for clarification of data.

## Results

### Search results

**Fig. 1:**
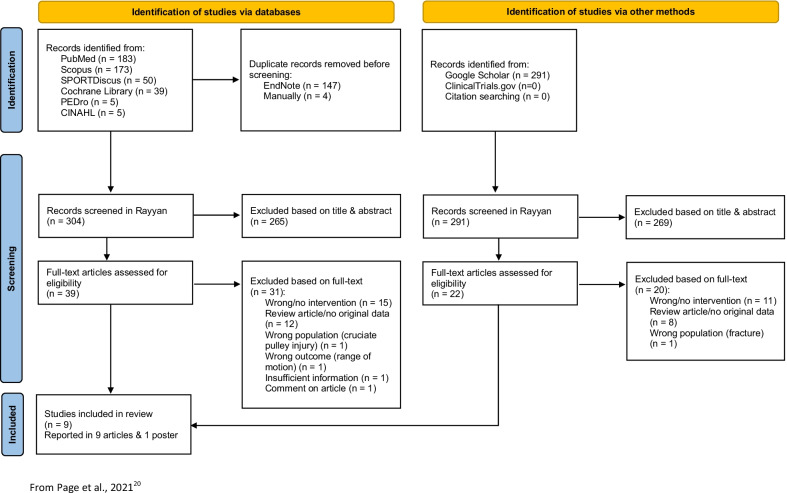
PRISMA 2020 flow diagram for systematic reviews, including searches of databases and other sources.From Page et al. [20]

The search process generated 746 records, of which 595 remained after removing duplicates. After screening titles and abstracts for relevance, and when necessary assessing full text articles, 585 articles were excluded according to eligible criteria. Agreement among reviewers were substantial, with number of observed identical agreements between 97 and 98% (Cohen’s kappa ranged from 0.66 to 0.75). Searches of ongoing trials, and backward and forward citation searches of included studies and identified previous reviews, did not identify any relevant or additional records. In total, eight studies and one case report, reported in nine articles [6, 7, 9, 11, 12, 15, 17, 27, 28] and one poster [29] were included. Two articles, one in an English journal [11] and one in a German journal [12], reported on the same study, and their results were therefore analysed as one. The selection process is illustrated in Fig. 1.

### Characteristics of included studies

The included studies were, besides one from 1990, conducted between 2000 and 2019, three in the USA and six in Europe (Germany, Austria, Switzerland and the UK). All were published in English, with one also being published in German. The studies included a total of 206 rock climbers (135 of which had pulley injuries, of which 12 were considered healed at time of intervention) [7, 11, 12, 15, 17, 27, 28], four uninjured non-climbers [6] and 23 pairs of fresh-frozen cadaver hands [9, 29]. In the studies on living individuals age (mean/range) ranged from 18 to 58 years and in the cadaver studies age ranged from 20 to 98 years, with one study [28] not specifying age of the participants. A predominant part of the subjects were male (n = 176), a few female (n = 43), and one study [29] did not specify sex of the participants (n = 14).

Two studies measured maximum force (N) upon rupture of A2, while loading fingers in full crimp position [9, 29]; two measured force against A2, either directly [6] or indirectly [17], while loading fingers in full crimp position; one measured MVC of FDP while loading fingers in full crimp position [15]; one measured MVC of FDP, in uninjured climbers, while loading fingers in full crimp position [28]; one measured muscle activation of FDS and FDP, in uninjured climbers, while loading fingers in full crimp position [27]; two studies measured bowstringing of the finger flexor tendons (FDS/FDP), while loading fingers in full crimp position [6, 15]; and two studies looked at time to return to sport (RTS) and perceived pain after pulley-injury [7, 11, 12]. Of these, two followed its participants for six to 15 months [7, 11, 12] while the others measured outcome variables at time of intervention only. [6, 9, 15, 17, 27–29]

Detailed characteristics of included studies are presented in Table 2.

**Table 2:** Included studies, n = 9 (reported in nine articles and one poster)

References, country	Study design	Participants	Intervention	Comparison	Outcome measures	Results
Bollen [7], UK	Case report	n = 1 (rock climber with pulley injury and clinical presentation of bowstringing), age 20, male	Taping, base of finger	None	Time to return to sports (RTS); pain; bowstringing	n = 1 could RTS without pain nor loss of function at 4w and 6mo follow up, bowstringing remained unchanged
Dykes et al. [27], USA	Randomized crossover trial	n = 10 (uninjured rock climbers); age range 18–22; 10 men, no women	Circular taping; H-taping. Loading of FDS & FDP in open hand & full crimp on “distal phalanx wide” edge	No taping. Loading of FDS & FDP in open hand & full crimp on “distal phalanx wide” edge	Muscle activation of FDS & FDP, measured with EMG	No difference in muscle activation of FDS & FDP between taped & non-taped fingers (*p* = 0.07)
Niegl et al. [17], Austria	Crossover trial	n = 11 (uninjured rock climbers); mean age 25; 11 men, no women	Circular taping. Loading of right hand in full crimp on 15 mm edge	No taping. Loading of right hand in full crimp on 15 mm edge	Changes in joint angles of PIP/DIP. Used to deduct force reduction against A2 (%)	14° less PIP-flexion in taped compared to non-taped finger. 10° less DIP-hyperextension in taped compared to non-taped finger *(p* < 0.01)*. On this basis Niegl et al. assumed a reduction of force on A2 by 11%
Partner et al. [28], UK	Randomized crossover trial	n = 50 (uninjured rock climbers); age unspecified; 25 men, 25 women	H-taping. Loading of hands in full crimp with Jamar dynamometer	No taping. Loading of hands in full crimp with Jamar dynamometer	Finger strength (MVC), measured with Jamar plus digital dynamometer	No difference in MVC between taped & non-taped fingers (*p* = 0.92)
Schweizer [6], Switzerland	Crossover trial	n = 16 (fingers) on 4 uninjured individuals; 3 men (30, 30 & 58 years of age), 1 woman (30 years of age)	Circular taping (over A2, or distal end of proximal phalange). Loading of dig. 3 & 4 in full crimp on 22 mm edge	No taping. Loading of dig. 3 & 4 in full crimp on 22 mm edge	Bowstringing (mm); force absorbed by taping (N); force of bowstringing (N)	Taping over A2 decreased bowstringing by 0.05 mm (2.8%) (*p* = 0.61) & at end of proximal phalange by 0.75 mm (22%) *(p* < 0.01)*. [NB: we calculated p-values from available mean, SD & sample size.] Taping absorbed 41–46 N (11–12%) of force from bowstringing. Force of bowstringing could not be measured, since the test proved too painful to the participants, and had to be aborted
Schöffl et al. [15], Germany	Crossover trial	n = 12 (rock climbers with previous pulley injuries (> 1 year earlier), grade 1–3); mean age 36; 12 men, no women	Circular taping; 8-taping; H-taping. Loading of single finger in full crimp & open hand on 20 mm edge	No taping. Loading of single finger in full crimp & open hand on 20 mm edge	Bowstringing (mm); finger strength (MVC)	Bowstringing without tape 3.77 mm, with 8-taping 3.70 mm, with circular taping 3.59 mm, with H-taping 3.19 mm *(p* < 0.05)*. MVC in injured finger was 13% higher with H-taping compared to no taping in full crimp *(p* < 0.01)*. [NB: I. Schöffl et al. reports different values in their body text and table for MVC, but have explained in private correspondence that this is due to rounding of decimals, and that the true between group difference of MVC is 13%] Taping made no difference to MVC in open hand or uninjured finger
Schöffl et al. [11]/[12], Germany	Prospective cohort study	n = 122 (rock climbers with pulley injuries, grade 1–4); mean age 29; 110 men, 12 women	Immobilization (2w), functional training (2-4w) & circular taping (grade 1–2, 3mo) or protective orthosis & circular taping (grade 3, 6mo). Surgery (grade 4)	None	Time to return to sports (RTS); pain	n = 87–88 available to follow up. n = 73 (grade 1–3) could RTS at 3mo with no to minor pain (n = 6 continued taping > 12mo), n = 7 with persistent pain received corticosteroid injections *& n* = *1 proceeded to surgery*. n = 7 (grade 4) straight to surgery [NB: V. Schöffl et al. reports one extra surgery participant in their German publication compared to their English, highlighted in italics above]
Tufaro et al. [29], USA	Controlled clinical trial	n = 112 (fingers) on 14 pairs of fresh frozen cadaver hands); age range 50–98, sex not specified	H-taping. Loading of single fingertip in full crimp until rupture of A2 (partially torn & intact)	No taping. Loading of single fingertip until rupture of A2 (partially torn & intact)	Force at A2 rupture (N); bowstringing (mm), but only measured for un-taped comparison	No difference between taped & non-taped finger at pulley rupture (torn A2, *p* = 0.39 & intact A2 *p* = 0.69)
Warme and Brooks [9], USA	Randomized controlled trial	n = 72 (fingers) on 9 pairs of fresh frozen cadaver hands); age range 20–47; 4 men, 5 women	Circular taping. Loading of single fingertip in full crimp until rupture of A2	No taping. Loading of single fingertip until rupture of A2	Force at pulley rupture (N)	No difference between taped & non-taped finger at pulley rupture (*p* = 0.53)

*Statistically significant results in bold, *A2* second annular ligament/pulley, *dig*. digitorum manus, *DIP* distal interphalangeal joint, *EMG* electromyography, *FDP* flexor digitorum profundus, *FDS* flexor digitorum superficialis, *mo* months, *MVC* maximal voluntary contraction, *N* newton/force, *NB* nota bene, *PIP* proximal interphalangeal joint, *RTS* return to sports, *SD* standard deviation, *w* weeks

### Clinical relevance

Included studies had a Cochrane clinical relevance score ranging from zero to five (out of five), with a median value of two. Agreement among reviewers was substantial (87%, Cohen’s kappa 0.73). For total scores, see Table 3. A complete breakdown of the Cochrane scores are also available in “Appendix 2”.

**Table 3:** Cochrane scale for clinical relevance, higher scores better, see “Appendix 2” for details

References, country	Cochrane score
Bollen [7], UK	0/5
Dykes et al. [27], USA	3/5
Niegl et al. [17], Austria	5/5
Partner et al. [28], UK	2/5
Schweizer [6], Switzerland	4/5
Schöffl et al. [15], Germany	5/5
Schöffl et al. [11], Germany	2/5
Schöffl et al. [12], Germany	2/5
Tufaro et al. [29], USA	0/5
Warme and Brooks [9], USA	2/5

### Finger taping methods

Three different methods for finger taping (circular taping, 8-taping and H-taping) were identified in the included studies, and are presented in Table 4.

**Table 4:** Finger taping methods

Method	Description	Studies
Circular taping	1.3–2.0 cm wide non-elastic tape wrapped 3–4 times around the proximal phalange, either directly above the A2 or slightly distal of the A2/over the distal end of the proximal phalange	Dykes et al. [27], Niegl et al. [17], Schweizer [6], Schöffl et al. [15], Warme and Brooks [9]
8-taping	tape applied in an 8-shape crossing the PIP-joint on the palmar side	Schöffl et al. [11], [12]; [N.B. method described in Schöffl et al. [15]]
H-taping	10 cm long and 1.5 cm wide non-elastic tape cut lengthwise from both sides, leaving 1 cm intact in the middle, taking the shape of an “H”. The two proximal ends are wrapped around the distal part of the proximal phalange, after which the PIP-joint is flexed, then the two distal parts are wrapped around the proximal part of the middle phalange	Dykes et al. [27], Partner et al. [28], Schöffl et al. [15], Tufaro et al. [29]

### Summary of findings

Summary of findings for all outcomes, and certainty of the evidence according to GRADE, are presented in Table 5. Details for each outcome are also described below. When appropriate, bar charts have been used to illustrate the data.

**Table 5:** Summary of findings for taping versus no taping, pulley injuries, rock climbers

Outcomes	Without taping	With taping	Number of participants (studies)	Certainty of the evidence (GRADE)	Conclusion
Pain, after grade 1–3 pulley injuries	N/A	90–91% of rock climbers reported no to minor pain after three months	123 (1 cohort study [11, 12] & 1 case report [7])	2/4 (before adjustment) 1/4 (after adjustment) − 0.5 inconsistency − 0.5 imprecision	There is **very low** certainty of evidence that taping reduces pain after grade 1–3 pulley injuries
Time to RTS, after grade 1–3 pulley injuries	N/A	90–91% of rock climbers could RTS after 3 months	123 (1 cohort study [11, 12] & 1 case report [7])	2/4 (before adjustment) 1/4 (after adjustment) − 0.5 inconsistency − 0.5 imprecision	There is **very low** certainty of evidence that taping allows for RTS after 3 months after grade 1–3 pulley injuries
Bowstringing, at proximal phalange, in uninjured individuals & rock climbers with previous grade 1–3 pulley injuries	Bowstringing without tape ranged from 3.45 to 3.77 mm	Bowstringing was 15–22% lower with taping	16 (2 crossover trials [6, 15])	4/4 (before adjustment) 2.5/4 (after adjustment) − 1 serious risk of bias − 0.5 imprecision	There is **low to moderate** certainty of evidence that taping reduces bowstringing after grade 1–3 pulley injuries
		1 rock climber with clinical bowstringing saw no effect of taping	1 (1 case report [7])		
Shearing forces against A2, in uninjured rock climbers/individuals	N/A	Taping absorbed 11–12% of shearing forces against A2	15 (2 crossover trials [6, 17])	4/4 (before adjustment) 2/4 (after adjustment) − 1 serious risk of bias − 0.5 indirectness − 0.5 imprecision	There is **low** certainty of evidence that taping reduces shearing forces against A2
Maximum force at pulley rupture, in cadaver hands	Force at pulley rupture ranged from 153 N (50% pre-torn, subjects aged 50 to 98) to 569 N (intact, subjects aged 20 to 47, male)	There was no significant difference with taping	23 pairs of fresh frozen cadaver hands (1 RCT [9], 1 CCT [29])	4/4 (before adjustment) 1.5/4 (after adjustment) − 0.5 risk of bias − 0.5 inconsistency − 1 serious indirectness − 0.5 imprecision	Taping does not affect forces needed for pulley rupture, **very low to low** certainty of evidence
MVC, in rock climbers, with previous grade 1–3 pulley injuries	Reported as mean normalized finger strength in percentage of body weight	MVC in full crimp was 13% greater with taping; there was no significant difference for open hand	12 (1 crossover trial [15])	4/4 (before adjustment) 1/4 (after adjustment) − 2 very serious risk of bias − 0.5 inconsistency − 0.5 imprecision	There is **low** certainty of evidence that taping increases MVC in full crimp for rock climbers with previous grade 1–3 pulley injuries
			1 (1 case report [7])		
		1 rock climber with clinical bowstringing saw no decrease in MVC with taping			
MVC & muscle activation, in uninjured rock climbers	MVC, one hand, full crimp, 24 kg in Jamar dynamometer; muscle activation measured with EMG	There was no significant difference in MVC or muscle activation with taping	60 (2 randomized crossover trials [27, 28])	4/4 (before adjustment) 3/4 (after adjustment) − 0.5 risk of bias − 0.5 imprecision	There is **moderate** certainty of evidence that taping does not affect MVC nor muscle activation in uninjured rock climbers

GRADE Working Group grades of evidence, from Cochrane Effective Practice and Organisation of Care (EPOC) [30]

4/4, High certainty: This research provides a very good indication of the likely effect. The likelihood that the effect will be substantially different is low

3/4, Moderate certainty: This research provides a good indication of the likely effect. The likelihood that the effect will be substantially different is moderate

2/4, Low certainty: This research provides some indication of the likely effect. However, the likelihood that it will be substantially different is high

1/4, Very low certainty: This research does not provide a reliable indication of the likely effect. The likelihood that the effect will be substantially different is very high

*CCT* controlled clinical trial, *EMG* electromyography, *GRADE* Grading of Recommendations Assessment, Development and Evaluation, *mm* millimetre, *MVC* maximum voluntary contraction, *N* Newton, *N/A* not applicable, *RCT* randomized controlled trial, *RTS* return to sports

### Effect of finger taping on function

Function (functional rating scales) was not measured in any of the included studies.

### Effect of finger taping on pain, after grade 1–3 pulley injury

After taping with figure-8 tape, or taping at base of finger, 90–91% of rock climbers, with pulley injuries (grade 1–3), reported no to minor pain at follow up at one or three months. The other 9–10% reported persistent pain, and were later treated with corticosteroid injections, with one climber needing reconstructive surgery [7, 11, 12]. Certainty of this evidence was graded as very low.

### Effect of finger taping on time to RTS, after grade 1–3 pulley injury

Taping with figure-8 tape or taping at base of finger allowed for return to sports after 3 months in 90–91% of rock climbers, with pulley injuries (grade 1–3). Of these, 7% needed to continue taping for > 12 months. [7, 11, 12] Certainty of this evidence was graded as very low.

### Effect of finger taping on bowstringing, after grade 1–3 pulley injury

**Fig. 2:**
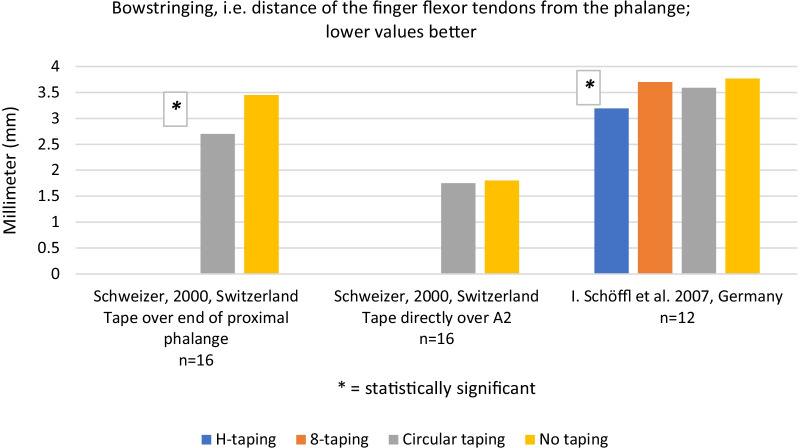
Bowstringing. NB: For Schweizer we calculated p-values from available mean, SD, sample size

Bowstringing without tape ranged from 3.45 to 3.77 mm at the proximal phalange. Taping with circular tape or H-tape decreased bowstringing by 15–22% compared to no taping (*p* < 0.05) [6, 15]. One case report [7] saw no effect on bowstringing, in a climber presenting with clinical bowstringing and taping at base of finger, neither at four weeks nor six months follow up. Certainty of this evidence was graded as low to moderate. Results, excluding Bollen [7], are presented in Fig. 2.

### Effect of finger taping on shearing forces against A2, in uninjured rock climbers

Taping with circular tape decreased shearing forces on A2 by 11–12% compared to no taping (*p* < 0.01) [6, 17]. Certainty of this evidence was graded as low.

### Effect of finger taping on pulley ruptures, in cadaver hands

**Fig. 3:**
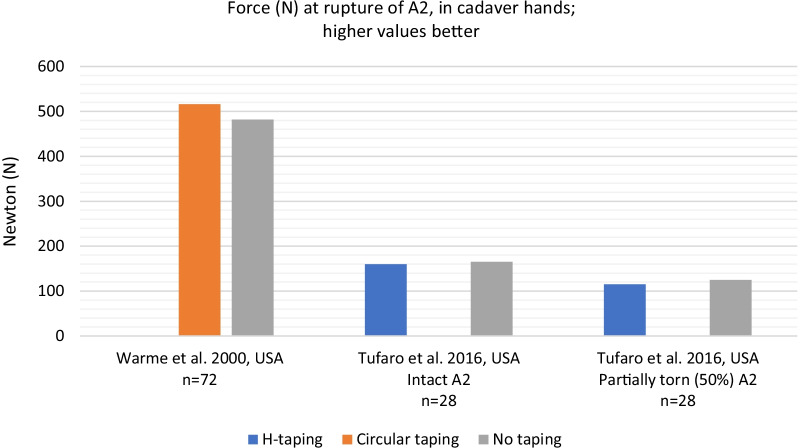
Force (N) at rupture of A2, in cadaver hands. NB: Values for Tufaro et al. are estimated based on visual presentation of data without exact numerical values in original source

Force at pulley rupture ranged from 153 N (for 50% pre-torn ligaments and subjects aged 50–98 years of age) to 569 N (with intact ligaments and all male subjects aged 20–47 years of age). There was no difference in maximum force at pulley rupture between circular tape or H-tape and no tape (*p* > 0.05) [9, 29]. Certainty of this evidence was graded as very low to low. Results are presented in Fig. 3.

### Effect of finger taping on MVC, after grade 1–3 pulley injury

MVC was reported as mean normalised finger strength in percentage of body weight. Taping with H-tape increased MVC by 13%, while single finger full crimping, in rock climbers with previous pulley injuries (grade 1–3) compared to no taping (*p* = 0.01). For open hand grip there was no difference in MVC [15]. One case report [7] saw no decrease in MVC four weeks after pulley injury, in a rock climber presenting with clinical bowstringing and taping at base of finger. Certainty of this evidence was graded as low.

### Effect of finger taping on MVC and muscle activation, in uninjured rock climbers

MVC was measured with Jamar dynamometer, one hand in full crimp, and muscle activation with electromyography (EMG). Taping with circular tape or H-tape did not increase MVC (24 kg reported in both groups) or muscle activation, compared to no taping [27, 28]. Certainty of this evidence was graded as moderate.

### Negative outcomes

No included studies reported any negative outcomes, or other side effects, of finger taping.

## Discussion

Based on nine studies, including 206 rock climbers (135 of which had pulley injuries), four uninjured non-climbers and 23 pairs of fresh-frozen cadaver hands, it is uncertain whether taping reduces pain or allows for a faster return to sports. It is also uncertain whether taping protects against complete pulley ruptures, decreases the sharing forces against the pulleys or increase MVC in full crimp. It is, on the other hand, likely that taping reduces bowstringing. Most likely, taping has no effect on open hand grip, or on MVC or muscle activation in uninjured rock climbers.

The clinical relevance of the included studies was very varied (ranging from zero to five) and the certainty of evidence ranged from very low to moderate. The general low certainty of evidence was mainly due to study design (including lack of control groups, lack of randomization and lack of blinding) as well as low sample sizes, with five studies [6, 7, 15, 17, 27] including 12 participants or less, and two studies [9, 29] being carried out on 14 pairs of fresh frozen cadaver hands or less. With the exception of Warme and Brooks [9], no power calculations were performed, although Dykes et al. [27] performed a post-hoc analysis and found their sample size to be considered small. The small sample sizes were thus generally considered a major risk of bias. Lack of presentation of sufficient data and statistical analyses (e.g. missing mean and *p* values for main outcomes), as well as general low level of evidence from observational studies and case reports were a further risk of bias affecting the overall certainty of evidence.

While the results from the cadaver studies are interesting in themselves, it is not self-evident that the results can be extrapolated to living individuals in general, and rock climbers in particular. Cadaver studies are established as a valid method within biomechanical research [31], but we should not a priori assume that dead tissue possesses the same properties as living tissue, and especially not living tissue that has been conditioned through years of climbing specific training. So, before corroborating evidence from living individuals and rock climbers, these results should be approached with caution.

With this caveat, we found that the cadaver studies fulfilled eligible criteria for inclusion in this review. One of them [9] was also included in the other systematic review on the same topic [16], and is frequently referred to by subsequent articles studying pulley injuries in rock climbers, including most studies in our own review [11, 12, 15, 17, 27, 29]. The cadaver studies also try to answer, in this aspect, a most interesting question. Namely, by directly testing the actual breaking point of annular ligaments, does taping increase the forces needed for complete rupture? An experiment that, for obvious ethical reasons, is impossible in vivo.

Another interesting finding in the cadaver studies were the big difference in force needed for rupture of the pulley-ligaments, 482–516 N and 153–190 N respectively. Most likely a result of the big age difference between the study samples, 20–47 years and 50–98 years respectively, since degeneration and decreased tensile strength of ligaments with age are well-established facts [32].

Also worthy of note is that the A2-pulley has been demonstrated to withstand loads of just above 400 N [33]. Schweizer’s study [6] also demonstrated that rock climbers habitually load the A2-pulley with forces up to 380 N and Bollen [7] demonstrated that a 70 kg rock climber that loses balance might chock-load a finger with forces of up to 450 N. A decrease of shearing force on the A2-pulley by 11–12% (41–46 N), as seen in Schweizer [6] and Niegl et al. [17], might then very well be the difference between a complete rupture and not, especially in an already injured ligament during rehabilitation. A decrease by 11–12% might also allow for earlier resistance training during rehabilitation, increased intensity during training/rehabilitation and/or an earlier return to sports. Further research is needed to corroborate these speculations.

Schöffl et al. [11, 12] observed a return to sports after three months in 90–91% of rock climbers, with pulley injuries grade 1–3. This corresponds with Bollen’s case report [7], and what has been observed in other studies [3], this timeframe might therefore be used as guidance for prognosis of conservatively treated pulley injuries (grade 1–3) in rock climbers. But since both Schöffl et al. [11, 12] and Bollen [7] lacked control groups (as well as had a general high risk of bias), a natural, intervention-independent, recovery process cannot be ruled out.

A limitation of this review was the broad PICO inclusion criteria, which produced a rather heterogeneous material, and made direct comparisons more difficult, including a meta-analysis of the data. A further limitation was the inclusion of non-RCTs, but excluding non-RCTs would have made a synthesis impossible due to lack of data, and was therefore deemed necessary. The lack of RCTs is clearly seen in the general low level of evidence for the different outcomes. Another limitation was that the search process was not peer reviewed prior to execution.

A strength of this review was the extensive and comprehensive literature search (seven databases searched, including grey literature), and almost 600 records screened for inclusion, with no exclusions being made neither for date nor language of publication. This makes it unlikely that any available records were missed. Scrutiny of reference lists of included studies and identified previous reviews and overviews also produces no new records, which is seen as an indicator of an exhaustive search strategy [34].

We would also like to add that, independent of our results, taping or not should always be seen as an adjuvant to any rehab protocol for pulley injuries in rock climbers. Main focus should always be, as with most injuries, progressive tissue loading through exercise therapy [11, 12, 35].

We would also like to highlight a recently published case series by Scheibler et al. [36] in June of 2021, presenting 12 patients (11 climbers) with triple pulley injuries (A2-A3-A4). All initially treated conservatively, except two who were not treated at all (whose injuries were later accidental findings). Conservative treatment consisted of thermoplastic pulley-protection splints and extension splints for two months, then climbing (open hand) could be resumed, using tape. The crimp grip was avoided for five to six months in total, after which all but two regained previous climbing level. The two who did not (both were diagnosed and treated late, more than two months after injury) underwent secondary reconstructive surgery, with good results. While these findings in themselves cannot lead to any definite conclusions, they might highlight yet another area for future research.

In summary, evidence of low to moderate certainty suggests that taping might reduce bowstringing by 15–22%. Evidence regarding pain, RTS, shearing forces against A2, pulley ruptures and MVC were all regarded as “very low”, “very low to low” or “low”, and were thus not considered reliable. Evidence of moderate certainty suggests that taping has no effect on MVC or muscle activation in uninjured climbers. Taping also had no effect on open hand grip in pulley-injured rock climbers. No adverse effects of taping were reported in the included studies. Due to the general low level of evidence, no definite recommendations for treatment using tape can be given, and future research on the possible effects are needed. As highlighted by Lum and Park [3], it is advisable that this research should include time to return to sports (RTS) as a primary outcome measure, since this is likely the most important outcome to rock climbers, as well as one that is often overlooked.

## Conclusions

Low to moderate evidence suggests that taping might reduce bowstringing of the finger flexor tendons. Moderate evidence suggests that taping has no effect on MVC or muscle activation in uninjured climbers. For other outcomes more studies evaluating the effects of taping are needed.

## Data Availability

The data and material analysed in the current review are available from the original source articles, or from the corresponding author upon reasonable request.

## Appendix 2: Cochrane scale for clinical relevance (from Furlan et al. [23])

**Table:**

References, country	Are the patients described in detail so that you can decide whether they are comparable to those that you see in your practice?^a^	Are the interventions and treatment settings described well enough so that you can provide the same for your patients?	Were all clinically relevant outcomes measured and reported?	Is the size of the effect clinically important?^b^	Are the likely treatment benefits worth the potential harms?	Total score (05)
Bollen [7], UK	No	No	No	No	Insufficient information	0/5
Dykes et al. [27], USA	Yes	Yes	Yes	No	No	3/5
Niegl et al. [17], Austria	Yes	Yes	Yes	Yes	Yes	5/5
Partner et al. [28], UK	No	Yes	Yes	No	No	2/5
Schweizer [6], Switzerland	Yes	Yes	No	Yes	Yes	4/5
Schöffl et al. [15], Germany	Yes	Yes	Yes	Yes	Yes	5/5
Schöffl et al. [11], Germany	Yes	No	No	No	Yes	2/5
Schöffl et al. [12], Germany	Yes	No	No	No	Yes	2/5
Tufaro et al. [29], USA	No	No	No	No	No	0/5
Warme and Brooks [9], USA	No	Yes	Yes	No	No	2/5

^a^Studies on cadavers automatically lost one point due to not being directly comparable to living individuals seen in practice

^b^A mean difference of < 10% was considered a small effect size; 10–20% medium; and > 20% = big (Cohen [24])

## Abbreviations

A1-5: Annular ligament/pulley 1–5
C1-3: Cruciate ligament/pulley 1–3
CCT: Controlled clinical trial
Dig.: Digitorum manus
DIP: Distal interphalangeal joint
EMG: Electromyography
FDP: Flexor digitorum profundus
FDS: Flexor digitorum superficialis
kg: Kilogram
MCID: Minimal clinically important difference
MCP: Metacarpophalangeal joint
mm: Millimetre
MVC: Maximum voluntary contraction
N: Newton/force
PIP: Proximal interphalangeal joint
RCT: Randomized controlled trial
RTS: Return to sport
SD: Standard deviation

## References

[1] Nelson NG, McKenzie LB (2009). Rock climbing injuries treated in emergency departments in the U.S., 1990–2007. Am J Prev Med.

[2] Lutter C, El-Sheikh Y, Schöffl I, Schöffl V (2017). Sport climbing: medical considerations for this new Olympic discipline. Br J Sports Med.

[3] Lum ZC, Park L (2019). Rock climbing injuries and time to return to sport in the recreational climber. J Orthop.

[4] Schöffl V, Popp D, Küpper T, Schöffl I (2015). Injury trends in rock climbers: evaluation of a case series of 911 injuries between 2009 and 2012. Wilderness Environ Med.

[5] Crowley TP (2012). The flexor tendon pulley system and rock climbing. JHAM.

[6] Schweizer A (2000). Biomechanical effectiveness of taping the A2 pulley in rock climbers. J Hand Surg Am.

[7] Bollen SR (1990). Injury to the A2 pulley in rock climbers. J Hand Surg Am.

[8] Schöffl I, Oppelt K, Jüngert J, Schweizer A, Neuhuber W, Schöffl V (2009). The influence of the crimp and slope grip position on the finger pulley system. J Biomech.

[9] Warme WJ, Brooks D (2000). The effect of circumferential taping on flexor tendon pulley failure in rock climbers. Am J Sports Med.

[10] Vigouroux L, Quaine F, Labarre-Vila A, Moutet F (2006). Estimation of finger muscle tendon tensions and pulley forces during specific sport-climbing grip techniques. J Biomech.

[11] Schöffl V, Hochholzer T, Winkelmann HP, Strecker W (2003). Pulley injuries in rock climbers. Wilderness Environ Med.

[12] Schöffl V, Hochholzer T, Winkelmann HP, Strecker W (2004). Zur Therapie von Ringbandverletzungen bei Sportkletterern. Handchir Mikrochir Plast Chir.

[13] Schneeberger M, Schweizer A (2016). Pulley ruptures in rock climbers: outcome of conservative treatment with the pulley-protection splint—a series of 47 cases. Wilderness Environ Med.

[14] Bellevue KD, Allan CH, Warme WJ (2018). The Novel Semilunar Pulley Orthosis (SPOrt) decreases flexor tendon-phalanx distance in climbers with chronic A2 pulley ruptures. Discoveries 2018.

[15] Schöffl I, Einwag F, Strecker W, Hennig F, Schöffl V (2007). Impact of taping after finger flexor tendon pulley ruptures in rock climbers. J Appl Biomech.

[16] Piculin J, Kacin A (2019). Vpliv togih lepilnih trakov na obremenitev krožnih vezi prstov pri plezalcih – sistematični pregled literature. Fizioterapija.

[17] Niegl G, Fuss FK, Tan MA, Moritz EF, Haake S (2006). Mechanical influence of finger taping in sport climbing. The engineering of sport 6.

[18] Huang X, Lin J, Demner-Fushman D. Evaluation of PICO as a knowledge representation for clinical questions. In: AMIA ... annual symposium proceedings. AMIA symposium, 2006; 2006. p. 359–63. PMC1839740 17238363

[19] Schardt C, Adams MB, Owens T, Keitz S, Fontelo P (2007). Utilization of the PICO framework to improve searching PubMed for clinical questions. BMC Med Inform Decis Mak.

[20] Page MJ, McKenzie JE, Bossuyt PM, Boutron I, Hoffmann TC, Mulrow CD (2021). The PRISMA 2020 statement: an updated guideline for reporting systematic reviews. BMJ (Clin Res Ed).

[21] Ouzzani M, Hammady H, Fedorowicz Z, Elmagarmid A (2016). Rayyan: a web and mobile app for systematic reviews. Syst Rev.

[22] EndNote X9 [Computer program]. Version X9.3.3. Philadelphia: Clarivate Analytics, The EndNote Team; 2013.

[23] Furlan AD, Pennick V, Bombardier C, van Tulder M (2009). updated method guidelines for systematic reviews in the Cochrane Back review group. Spine (Phila Pa 1976).

[24] Cohen J (1988). Statistical power analysis for the behavioral sciences.

[25] PEDro physiotherapy evidence database. Sydney, Australia. [cited 8 March 2021]. https://www.pedro.org.au

[26] Guyatt GH, Oxman AD, Kunz R, Vist GE, Falck-Ytter Y, Schunemann HJ (2008). What is “quality of evidence” and why is it important to clinicians?. BMJ.

[27] Dykes B, Johnson J, San Juan JG (2019). Effects of finger taping on forearm muscle activation in rock climbers. J Electromyogr Kinesiol.

[28] Partner R, Fox G, Francis P, Jones G (2018). Does taping of the annular pulleys of the fingers improve grip strength in climbers?. J Phys Educ Sport.

[29] Tufaro R, Telis A, Larson D, Mercer D, Salas C. The H-taping method for prophylactic or temporary treatment of partial A2 pulley tears during rock climbing: a biomechanical study. Poster presented at: ORS 2016 annual meeting; Mar 5–8 2016; Orlando, FL; 2016.

[30] Cochrane Effective Practice and Organisation of Care (EPOC). EPOC worksheets for preparing a Summary of Findings (SoF) table using GRADE. EPOC Resources for review authors, 2017. epoc.cochrane.org/resources/epoc-resources-review-authors. Accessed 2nd Oct 2021.

[31] Girardi BL, Attia T, Backstein D, Safir O, Willett TL, Kuzyk PR (2016). Biomechanical comparison of the human cadaveric pelvis with a fourth generation composite model. J Biomech.

[32] Buckwalter JA, Woo SLY (1996). Age-related changes in ligaments and joint capsules: implications for participation in sports. Sports Med Arthrosc Rev.

[33] Lin GT, Cooney WP, Amadio PC, An KN (1990). Mechanical properties of human pulleys. J Hand Surg Am.

[34] Bramer WM, de Jonge GB, Rethlefsen ML, Mast F, Kleijnen J (2018). A systematic approach to searching: an efficient and complete method to develop literature searches. J Med Libr Assoc.

[35] Schöffl V, Schöffl I (2006). Injuries to the finger flexor pulley system in rock climbers: current concepts. J Hand Surg Am.

[36] Scheibler AG, Janig C, Schweizer A (2021). Primarily conservative treatment for triple (A2–A3–A4) finger flexor tendon pulley disruption. Hand Surg Rehabil.

